# 3D spatial organization and improved antibiotic treatment of a *Pseudomonas aeruginosa–Staphylococcus aureus* wound biofilm by nanoparticle enzyme delivery

**DOI:** 10.3389/fmicb.2022.959156

**Published:** 2022-11-16

**Authors:** Alba Rubio-Canalejas, Aida Baelo, Sara Herbera, Núria Blanco-Cabra, Marija Vukomanovic, Eduard Torrents

**Affiliations:** ^1^Bacterial Infections and Antimicrobial Therapies Group, Institute for Bioengineering of Catalonia, The Barcelona Institute of Science and Technology, Barcelona, Spain; ^2^Microbiology Section, Department of Genetics, Microbiology and Statistics, Faculty of Biology, University of Barcelona, Barcelona, Spain; ^3^Advanced Materials Department, Institute Jozef Stefan, Ljubljana, Slovenia

**Keywords:** biofilm, wound healing, chronic infection, antimicrobial therapies, nanoparticle

## Abstract

Chronic wounds infected by *Pseudomonas aeruginosa* and *Staphylococcus aureus* are a relevant health problem worldwide because these pathogens grow embedded in a network of polysaccharides, proteins, lipids, and extracellular DNA, named biofilm, that hinders the transport of antibiotics and increases their antimicrobial tolerance. It is necessary to investigate therapies that improve the penetrability and efficacy of antibiotics. In this context, our main objectives were to study the relationship between *P. aeruginosa* and *S. aureus* and how their relationship can affect the antimicrobial treatment and investigate whether functionalized silver nanoparticles can improve the antibiotic therapy. We used an optimized *in vitro* wound model that mimics an *in vivo* wound to co-culture *P. aeruginosa* and *S. aureus* biofilm. The *in vitro* wound biofilm was treated with antimicrobial combinatory therapies composed of antibiotics (gentamycin and ciprofloxacin) and biofilm-dispersing free or silver nanoparticles functionalized with enzymes (α-amylase, cellulase, DNase I, or proteinase K) to study their antibiofilm efficacy. The interaction and colocalization of *P. aeruginosa* and *S. aureus* in a wound-like biofilm were examined and detailed characterized by confocal and electronic microscopy. We demonstrated that antibiotic monotherapy is inefficient as it differentially affects the two bacterial species in the mixed biofilm, driving *P. aeruginosa* to overcome *S. aureus* when using ciprofloxacin and the contrary when using gentamicin. In contrast, dual-antibiotic therapy efficiently reduces both species while maintaining a balanced population. In addition, DNase I nanoparticle treatment had a potent antibiofilm effect, decreasing *P. aeruginosa* and *S. aureus* viability to 0.017 and 7.7%, respectively, in combined antibiotics. The results showed that using nanoparticles functionalized with DNase I enhanced the antimicrobial treatment, decreasing the bacterial viability more than using the antibiotics alone. The enzymes α-amylase and cellulase showed some antibiofilm effect but were less effective compared to the DNase I treatment. Proteinase K showed insignificant antibiofilm effect. Finally, we proposed a three-dimensional colocalization model consisting of *S. aureus* aggregates within the biofilm structure, which could be associated with the low efficacy of antibiofilm treatments on bacteria. Thus, designing a clinical treatment that combines antibiofilm enzymes and antibiotics may be essential to eliminating chronic wound infections.

## Introduction

Wounds and wound management represent a relevant but sometimes unrecognized, growing challenge for healthcare and the economic system worldwide. Only wound management accounts for 3% of the total healthcare expenses such as materials, professionals, and hospitalization costs, among others (Lindholm and Searle, 2016). Wound features entail susceptibility to microbial colonization and proliferation, leading to infection and wound-healing difficulties (Zhao et al., 2016).

Commonly, infected wounds have an acute or a chronic etiology depending on their healing time frame and healing capacity (Zhao et al., 2016). However, a remarkable 80% of chronic wounds contain biofilms (Malone et al., 2017), and 1.5–2 million people in Europe and around 6.5 million people in the USA are affected by this condition (Lindholm and Searle, 2016). Many dermal infections, including surgical, burn, or bite wound infections and other skin abrasions, are classified as acute and are solved in a timely fashion. Nonetheless, some patients suffer from pathologic conditions such as diabetes, obesity, immunosuppression, or drug treatments, which predispose the delayed or failed healing of infected wounds. Foot, leg, and decubitus ulcers are the most representative examples of chronic infections (Zhao et al., 2016). Indeed, chronic wounds are also associated with a persistent host inflammatory state and an incomplete antimicrobial response against treatments (Zhao et al., 2016).

Free bacteria irreversibly attach to the surface of wounds and rapidly divide and recruit other microorganisms to form microcolonies (Fleming and Rumbaugh, 2017), a process regulated by environmental or physiological cues (Liu et al., 2016). Eventually, microcolonies evolve into a tridimensional, generally polymicrobial community known as biofilm (Liu et al., 2019). Biofilm presence in wounds is associated with chronicity and wound complications. Nevertheless, proper biofilm-based wound care and management strategies can minimize wound-healing impact in our society. Currently, wound debridement, systemic antibiotics, biocides, and antibiofilm agents are the main strategies against wound biofilms (Hrynyshyn et al., 2022). However, it is imperative to develop new effective strategies that are tested prospectively and employed in chronic wounds to support the healing process and reduce infection rates (Percival et al., 2012).

*Pseudomonas aeruginosa* and *Staphylococcus aureus* are two ubiquitous opportunistic pathogens most frequently isolated from biofilm-based chronic infections (DeLeon et al., 2014). These bacteria have a complex relationship in nature where they can show a competitive interplay as the exoproducts produced by *P. aeruginosa* suppress *S. aureus* growth and even provoke the appearance of small colony variants in the staphylococcal population (Kumar and Ting, 2015; Cendra et al., 2019; Cendra and Torrents, 2021). However, the polymicrobial nature of most biofilms allows bacteria to exploit synergistic relationships and therefore favors their cooperation and virulent traits (Dalton et al., 2011). Interestingly, the dual infection of these bacteria increases their resistance and/or tolerance to antimicrobials, prevalence, tissue colonization, and their ability to delay healing (DeLeon et al., 2014; Brothers et al., 2015).

Bacteria in biofilms are protected by a self-synthesized extracellular polymeric matrix formed by polysaccharides, proteins, lipids, and extracellular DNA (eDNA), commonly called extracellular polymeric substance (EPS) (Fleming and Rumbaugh, 2017). The encapsulation of bacteria inside the biofilm provides the acquisition of new features different from their planktonic states, such as a dormant state, the host immune system avoidance, or resistance/tolerance to some antimicrobial treatments. The biofilm is a mechanical barrier against endogenous and exogenous antimicrobial strategies that impedes the reepithelization process (Fleming et al., 2017).

Notwithstanding, mature biofilms are dynamic. The bacteria forming the biofilm can actively modify it during their lifecycle in response to environmental changes (i.e., nutrient starvation and oxygen depletion, accumulation of toxic products, immune system challenges, quorum sensing, and others) (Fleming and Rumbaugh, 2017). Eventually, planktonic cells are released from the biofilm and migrate to more favorable environments, always promoting bacterial survival (Fleming and Rumbaugh, 2017). In nature, the dispersion of bacteria can be accomplished through the segregation of bacterial biofilm-dispersing enzymes that act on the EPS. Some studies have already evaluated the capacity of various biofilm-dispersing enzymes such as glycolytic hydrolases, proteases, and DNases to increase biofilm disaggregation and the number of free bacteria (Tetz and Tetz, 2010; Fleming et al., 2017). Interestingly, the use of biofilm-dispersing enzymes is a potential pre-treatment as they increase the susceptibility of the biofilm-forming bacteria (Redman et al., 2021). In addition, the dual infection of chronic wounds with *P. aeruginosa* and *S. aureus* in the clinical setting shows the importance of using a combinatory therapy of antibiotics to target both bacterial pathogens (Serra et al., 2015). The antibiotic combination may depend on the patient and the clinical outcome of the chronic wound. There are treatments with different classes of antibiotics, such as penicillin, cephalosporins, aminoglycosides, and fluoroquinolones (Garner et al., 2020).

Due to the complexity of the biofilm and the wound-healing process, it is difficult to find an *in vivo* or *in vitro* model that appropriately describes the biofilm's behavior, physiology, and microenvironment in the skin (Klein et al., 2018). Here, we use an optimized wound-like biofilm *in vitro* model previously described (Sun et al., 2008; DeLeon et al., 2014) that allows *P. aeruginosa* and *S. aureus* co-culture in the wound environment, similar to *in vivo* conditions.

In this study, we used a convenient and reliable *in vitro* wound-like biofilm model to evaluate the efficacy of different antibiofilm strategies. We used four biofilm-dispersing enzymatic treatments combined with antibiotics to test their effect on bacterial viability. Also, we aim to better understand the *P. aeruginosa* and *S. aureus* synergistic relationship by studying their interactions and colocalization inside the biofilm.

## Materials and methods

### Bacterial strains and growth conditions

*Pseudomonas aeruginosa* PAO1 (ATCC 15692) and *Staphylococcus aureus* SA31 (ATCC 29213) were maintained in initial cryo-stock cultures at −80°C, and they resuscitated in Luria-Bertani (LB) agar (Scharlab, S.L., Spain) and Tryptic Soy Agar (TSA) (Scharlab, S.L., Spain) plates, respectively. Isolated colonies were grown overnight in LB broth and Tryptic Soy Broth (TSB) (Scharlab, S.L., Spain), respectively, at 37°C with shaking at 200 rpm. Unless otherwise specified, all reagents were purchased from Sigma, Spain.

### Antibiotics and matrix-degrading enzymes

The antibiotics gentamicin sulfate (Gm) (PanReac AppliChem, Spain) and ciprofloxacin hydrochloride (Cip) (Cayman Chemical, USA) were employed simultaneously at a final concentration of 13.5 μg/ml according to the values shown in Supplementary Figure S1. α-amylase from *Bacillus subtilis* (MP Biomedicals, USA), cellulase from *Aspergillus niger* (MP Biomedicals, USA), deoxyribonuclease I from bovine pancreas (DNase I), and proteinase K from *Tritirachium album* (PanReac AppliChem, Spain) were used as biofilm-dispersing enzymes at different concentrations. The enzymes were tested alone or with gentamicin and ciprofloxacin.

### Antibacterial susceptibility testing and MIC_50_

*P. aeruginosa* PAO1 and *S. aureus* SA31 grew on LB and TSB, respectively, until they reached an optical density (OD_550_ nm) of 0.1, which corresponds to 10^7^ CFU/ml for each bacterial species. The bacteria were plated in a microtiter plate (Corning 3596 Polystyrene Flat Bottom 96 Well, Corning, USA) using several concentrations of gentamicin (from 0.0613 to 64 μg/ml), ciprofloxacin (from 0.0613 to 64 μg/ml), or silver nanoparticles (AgNP, from 0.03125 to 1 mg/ml). Bacterial growth at 37°C and 150 rpm was monitored for 16 h, measuring the absorbance at 550 nm every 15 min in a SPARK Multimode microplate reader (Tecan, Switzerland). The minimum inhibitory concentration of 50% (MIC_50_) corresponds with the concentration of the antibiotics that inhibited bacterial growth by 50%.

### Nanoparticle synthesis and characterization

AgNP immobilized with enzymes were formed using α-amylase, cellulase, DNase I, and proteinase K. The nanoparticles were synthesized using the chemical reduction method (Kandarp Mavani, 2013). Different options were investigated to functionalize the enzymes on the nanoparticles. (1) Aqueous solutions of silver nitrate (AgNO_3_) (50 ml, 4 mM) and enzyme (25 ml, 0.4 mg/ml) were premixed for 10 min (200 rpm, 25°C). It was followed by the addition of sodium borohydride (NaBH_4_) (50 ml, 6 mM), which resulted in instant nanoparticle formation. (2) For forming bare AgNP, silver precursor was premixed with water following NaBH_4_ reduction. Reaction mixtures were kept for 2 h at 200 rpm and then centrifuged at 8,000 rpm for 20 min to collect formed AgNP. In the next step, AgNP were re-dispersed in fresh enzyme solutions in Tris-buffer (50 mM Tris-HCl, pH = 8) and mixed for 2 days (200 rpm, 25°C). The maximum enzymatic activity was obtained with the two-step approach. The resulting dispersions were precipitated by centrifugation at 8,000 rpm, washed in fresh Tris-HCl, frozen in liquid nitrogen, and freeze-dried. All syntheses were done using lab-made ultra-pure water (Purelab Option-Q7, ELGA, UK).

### Nanoparticle-enzyme activity quantification

The α-amylase and cellulase activities in the grafted AgNP were quantified by calculating the amount of reducing sugars produced in the polysaccharides' hydrolysis by DNS (3,5 dinitrosalicylic acid) method (Miller, 1959) following the protocol of Bernfeld (1955) for the α-amylase enzymatic activity and the protocol described by Wood and Bhat (1988) for the cellulase activity. Briefly, 10 mg/ml of starch was diluted in 400 μl of 0.02 M phosphate buffer and mixed with 400 μl of the α-amylase-AgNP for 3 min at 20°C. On the contrary, 20 mg/ml of sodium carboxymethyl cellulose (Acros Organics, Spain) diluted in 200 μl of 0.05 M citrate buffer was mixed with 200 μl of the cellulase-AgNP for 30 min at 50°C. Both reactions were stopped by adding the color reagent solution (1.06 M potassium sodium tartrate tetrahydrate and 43 mM DNS) and then boiled (400 μl of color reagent solution added and then boiled for 15 min in the α-amylase reaction, and 600 μl of color reagent solution added and then boiled for 5 min in the cellulase reaction). Standard curves of the reducing sugars produced in the reaction (maltose for α-amylase and glucose for cellulase) were performed by mixing different concentrations of the reducing sugars with the color reagent solution at the same volume ratio as with the AgNP. Colorimetric quantification of reducing sugars was performed by absorbance measurement at 540 nm. One unit is defined as the amount of enzyme which liberated 1 μmole of a reducing sugar per minute under the assay conditions.

To calculate the NP DNase I activity, 100 ng of *P. aeruginosa* genomic DNA was mixed with known concentrations of AgNP containing DNase I (up to 20 μl final volume). The mixtures were incubated for 30 min at 37°C, then loaded into a 0.8% agarose gel stained with ethidium bromide, and visualized under the UV light in a Gel Doc^TM^ XR+ (Bio-Rad Laboratories, Spain). The DNase I activity was calculated by quantifying DNA degradation with Quantity One (Bio-Rad Laboratories, Spain) software. One unit is defined as the enzyme that degrades 1 μg of DNA per hour.

Proteinase K-AgNP activity was determined by quantifying tyrosine released from denatured hemoglobin proteolysis by the Folin–Ciocalteu assay (Anson, 1938). Briefly, 2.5 ml of a hemoglobin substrate solution (2% hemoglobin and 6 M urea in 100 mM potassium phosphate buffer) was mixed with 0.5 ml of the Proteinase K-AgNP and incubated at 37°C for 10 min. The reaction was stopped by adding 5 ml of 5% trichloroacetic acid, and the solution was filtered through a 0.45 μm filter. About 1.5 ml of 1 N of Folin and Ciocalteu's phenol reagent was then added to 2.5 ml of the filtered product of the proteolysis reaction or the standard tyrosine solution (1.1 mM L-Tyr). The solutions were incubated for 30 min at room temperature, and the tyrosine was quantified by absorbance measurement at 750 nm. One unit of enzyme will produce 1 μmole of tyrosine per minute.

The commercial enzymes at known concentrations were used as a control to determine the activity of the enzyme-coated nanoparticles in each activity quantification assay.

### *In vitro* wound-like biofilm model

The wound-like medium (WLM) was made by combining 45% Bolton broth (Scharlab, S.L., Spain), 50% bovine plasma (VWR, Biowest SAS, France), and 5% lacked horse red blood cells (hemolyzed RBCs, Thermo Scientific Oxoid Ltd., UK) as described previously to a final 1 ml volume per WLB experiment (Sun et al., 2008; Dalton et al., 2011; DeLeon et al., 2014). About 1 ml of the WLM was placed in a 12 × 75 mm glass tube, inoculated with ~10^4^ CFU/mL of fresh *S. aureus* and *P. aeruginosa* cultures, and grown at 37°C for 24 h in static conditions until forming the wound-like biofilm clot.

### Wound-like biofilm antimicrobial assays

Antibiotics, enzymes, or nanoparticle-coated enzymes were freshly diluted in sterile 1x phosphate-buffered saline (PBS) (Fisher Scientific, Spain) to a final concentration of 13.5 μg/ml for gentamycin and ciprofloxacin, 20 and 40 μg/ml for α-amylase, 100 and 200 μg/ml for cellulase, 0.2 and 0.4 μg/ml for DNase I, 20 and 40 μg/ml for proteinase K, and 1 and 2 mg/ml for the AgNP. The wound-like biofilms (WLBs) were gently mixed avoiding biofilm disruption with 1 ml of the disaggregating solutions to be tested and left at 37°C for 16 h in static conditions. After incubation, WLBs were rinsed once with 1 ml of 1x PBS and weighted in an Entris Precision Balance (Sartorius, Goettingen, Germany). The WLBs were weighed inside a tube, and afterward, the weight of the tube was subtracted. Washed WLBs were resuspended with 1 ml of 1x PBS + 0.05% Tween20 (Sigma-Aldrich, Spain) and dispersed using a homogenizer T 10 basic ULTRA TURRAX (IKA-Werke GmbH & Co. KG, Germany) with an 8 mm diameter probe. The samples were diluted in PBS + 0.05% Tween 20, plated on *Staphylococcus* isolation agar [TSA containing 7.5% (w/v) of NaCl] and *Pseudomonas* isolation agar (LB agar containing 2 mg/ml of crystal violet), and incubated overnight at 37°C (Supplementary Figure S2). The total counts of each bacterial strain were used to determine the colony-forming units per gram of WLB clot (CFU/g).

### Wound-like biofilm staining and confocal microscopy imaging

The WLB clot was cut into three slices, top, middle, and bottom, to study the structure and spatial organization of *P. aeruginosa* and *S. aureus* inside the biofilm using the Bacterial Viability and Gram Stain Kit (Biotium, Fremont, USA). This kit combines the dye DAPI, which binds to the bacterial DNA, and the wheat germ agglutinin (WGA) coupled to a CFTM-488A fluorophore that binds the N-acetylglucosamine present in the peptidoglycan of gram-positive bacteria; thus, *P. aeruginosa* was stained with DAPI, and *S. aureus* was stained with DAPI and WGA. After staining, the samples were incubated for 60 min on ice. To discriminate between genomic bacterial DNA and eDNA, two dyes were used: SYTO60 (Thermo Scientific Oxoid Ltd., UK), which stains the genomic bacterial DNA, and TOTO-1 (Thermo Scientific Oxoid Ltd., UK), which stains the eDNA. The samples were incubated for 15 min on ice after staining. A Zeiss LSM 800 confocal laser scanning microscope (CSLM, Zeiss, Germany) was used to scan the samples. ImageJ and COMSTAT 2 software were used to measure clusters areas, analyze the images, and quantify eDNA (Heydorn et al., 2000).

### Scanning electron microscopy imaging of the clots

Field-emission scanning electron microscopy (FE-SEM) was used to visualize the structure and distributions of bacterial species in the mixed-species WLB. WLB clots were cut into slices and introduced into a 3% glutaraldehyde solution. After 3 h, the solution was replaced by fresh glutaraldehyde solution and incubated overnight at 4°C. Clot samples were subsequently washed three times with 1 × PBS, and dehydration was later performed by immersing the samples in increased ethanol concentrations (30, 50, 70, 90, and 100% v/v) for 30 min. The clots were afterward dried with a Critical Point Dryer (CPD BalTec 030, BalTec AG, Liechtenstein), covered with gold, and visualized using the field-emission scanning microscope Nova NanoSEM 230 (FEI Company, USA).

### Transmission electron microscopy

Transmission electron microscopy (TEM) investigation was performed at Tecnai Spirit 120 kV microscope. Nanoparticles were dispersed in water using an ultrasonic bath for a couple of minutes and deposited on copper lacey carbon grids.

### Statistical analysis

GraphPad Prism 9.0 (GraphPad Software, USA) was used to perform statistical analyses and generate histograms. Data values were expressed as the mean ± standard deviation. Significant differences between conditions were determined using unpaired Student's *t*-tests.

## Results

### Different antibiotic resistance of *P. aeruginosa* and *S. aureus* growing in a wound-like biofilm

**Figure 1:**
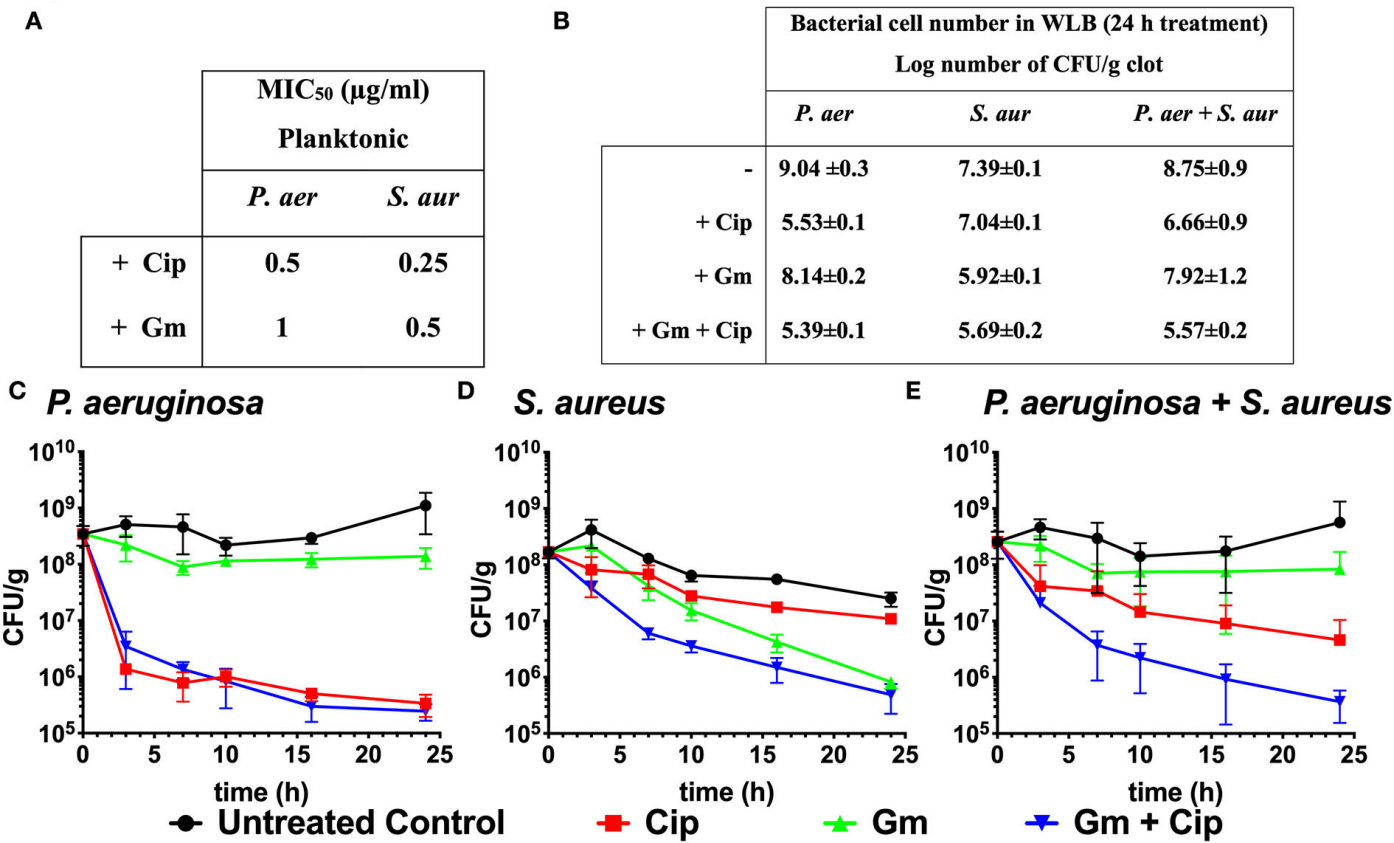
Bactericidal effect of ciprofloxacin and gentamicin. **(A)** MIC_50_ values of ciprofloxacin (Cip) and gentamicin (Gm) on *P. aeruginosa* (*P. aer*) and *S. aureus* (*S. aur*) in planktonic culture. **(B)** Log number of bacterial cell of *P. aeruginosa, S. aureus*, and total bacteria comparing treated (with ciprofloxacin and gentamicin at 13.5 μg/ml) to untreated samples after 24 h in a dual-species WLB. **(C–E)** Bacterial cell number in the clot (CFU/g) measured over 24 h without treatment, with ciprofloxacin, gentamicin, and gentamicin + ciprofloxacin at a final concentration of 13.5 μg/ml. **(C)** Cell number of *P. aeruginosa*, **(D)** *S. aureus*, and **(E)** total bacteria (*P. aeruginosa* and *S. aureus*).

The aminoglycoside gentamicin (Gm) and the fluoroquinolone ciprofloxacin (Cip) are broad-spectrum antibiotics commonly used to treat wounds containing *P. aeruginosa* and *S. aureus* infections (Serra et al., 2015; Negut et al., 2018). These antibiotics display high activity against non-resistant strains of both species. In this work, we determined the MIC_50_ values of gentamicin and ciprofloxacin in planktonic cultures of *P. aeruginosa* and *S. aureus* (Figure 1A). We used the previously described WLB model (see Materials and methods) that mimics an infected wound with a co-culture of *P. aeruginosa* and *S. aureus* to evaluate the local treatment efficacy of gentamicin and ciprofloxacin.

Although the *P. aeruginosa* and *S. aureus* monomicrobial planktonic cultures are susceptible to gentamicin and ciprofloxacin (MIC_50_ ranges from 0.25 to 1 μg/ml, Figure 1A), the biofilm structure found in the WLB confers protection to the bacteria, favoring the appearance of tolerance traits against antimicrobial treatments (Fazli et al., 2009). Figures 1B–E reflects the different bactericidal effects of gentamicin and ciprofloxacin when *P. aeruginosa* and *S. aureus* grew on polymicrobial WLB for 24 h. After 24 h, we observed that ciprofloxacin reduced *P. aeruginosa* cell number by 3.5 logs, while less than one log was reduced for *S. aureus*. The gentamicin treatment produced the opposite effect and reduced 1.5 log number for *S. aureus* and by less than a log for *P. aeruginosa* cells (Figures 1B–D). Considering both bacteria (*P. aeruginosa* plus *S. aureus*), the treatment with gentamicin reduced the total cell number by 0.83 logs, and ciprofloxacin reduced around 2.1 logs of the bacteria. Only when both antibiotics were used simultaneously, the whole cell number was reduced by more than 3.2 log number (Figures 1B,E).

### Immobilization and characterization of biofilm disaggregate silver nanoparticles

**Figure 2:**
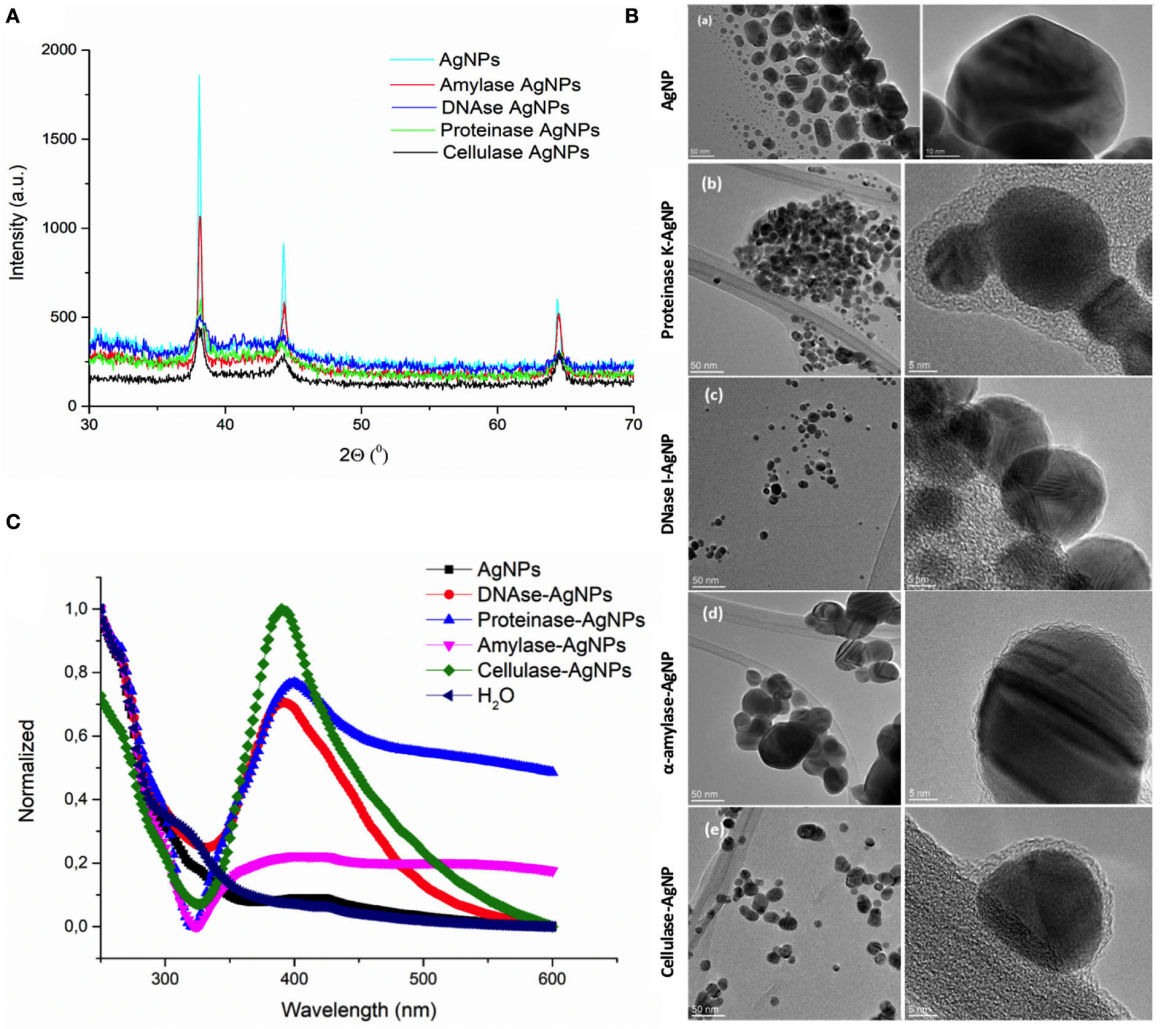
Characterization of silver nanoparticles (AgNP). **(A)** X-ray diffraction patterns of bare AgNP and AgNP with immobilized α-amylase, DNase I, proteinase K, and cellulase. **(B)** TEM images of AgNP with different surfaces: bare AgNP without enzyme (a), AgNP with proteinase K (b), DNase I (c), α-amylase (d), and cellulase (e). Images on the left present particle size and agglomeration, while those on the right look closely at the organic coating immobilized on their surface. **(C)** UV-vis spectra showing the stability of AgNP without and with different enzymes (DNase I, α-amylase, cellulase, and proteinase K) immobilized to their surface based on the presence or absence of surface plasmon resonance maximum (SPR), its position, and width.

To target the matrix of polymicrobial biofilms more effectively, we designed nanosized particles with enzymes (α-amylase, cellulase, DNase I, and proteinase K) immobilized on their surface (see specifications in Table 1). Along with the potential improvement in antibacterial treatment obtained by decomposing the matrix, the enzymes were meant to point toward the most relevant components that keep the stability of the matrix preventing the activity of antibiotics. Formed AgNP were nanospheres with cubic crystalline structure (JCPDS No. 4-0783) (Figures 2A,B). Without the enzyme, they were unstable aggregates with almost neutral zeta potential and high effective diameter (Table 1). These aggregates were formed of nanoparticles with broad size distribution (Figure 2B). Once the enzymes were immobilized to the AgNP surface, their stability improved significantly. It was observed an increase of up to 5.7 times in the magnitude of their zeta potentials and a significant reduction of up to 41 times in their effective diameters (Table 1). TEM analysis revealed that different enzymes protected particle growth and stability differently. The most effective were DNase I and cellulase, which kept the primary grains to a few tens of nanometers, while α-amylase and proteinase K protected AgNP from growing larger (Figure 2B). Another proof for immobilization-affected stability was the changes detected in AgNP optical spectra (Figure 2C). Due to intensive agglomeration, non-immobilized AgNP did not show any surface plasmon resonance maximum (SPR), which was detected for all the enzyme immobilized AgNP. The narrowest SPR, which shifted to lower wavelengths, was detected for the most stable DNase I and cellulase-protected AgNP. Although immobilization of the enzymes protected AgNP growth most effectively in the case of DNase I and cellulase, the activity of enzymes immobilized to their surface differs significantly. While DNase I immobilized to AgNP retained high enzymatic activity (276 U/mg NP), the activity obtained for cellulase on AgNP was much lower (0.15 U/mg NP) (see detailed characterization in Table 1). According to the highest morphological stability and enzymatic activity, it could be expected that AgNP-DNase I would be the most effective in disaggregating EPS matrix and in penetration inside its volume.

**Table 1:** Composition, encapsulation efficiency, and properties of drug-loaded nanoparticles.

	**Enzyme coupled**	**Enzyme activity (U/mg NP)**	**Effective diameter (nm)**	**Polydispersity index**	**Z-potential (mV)**
AgNP	–	–	1,172 ± 28	0.304 ± 0.025	−3.5 ± 9
AgNP-α-amylase	α-amylase	55.89	101.9 ± 1.0	0.320 ± 0.004	−17.1 ± 0.6
AgNP-Cellulase	Cellulase	0.15	28.4 ± 0.2	0.365 ± 0.004	−20.1 ± 1.4
AgNP-DNase I	DNase I	276	39.3 ± 0.2	0.233 ± 0.005	−16.1 ± 12
AgNP-Proteinase K	Proteinase K	0.37	356.1 ± 17	0.324 ± 0.009	−20.0 ± 1.5

### Silver nanoparticles have a different effect on bacteria in the wound-like biofilm

**Figure 3:**
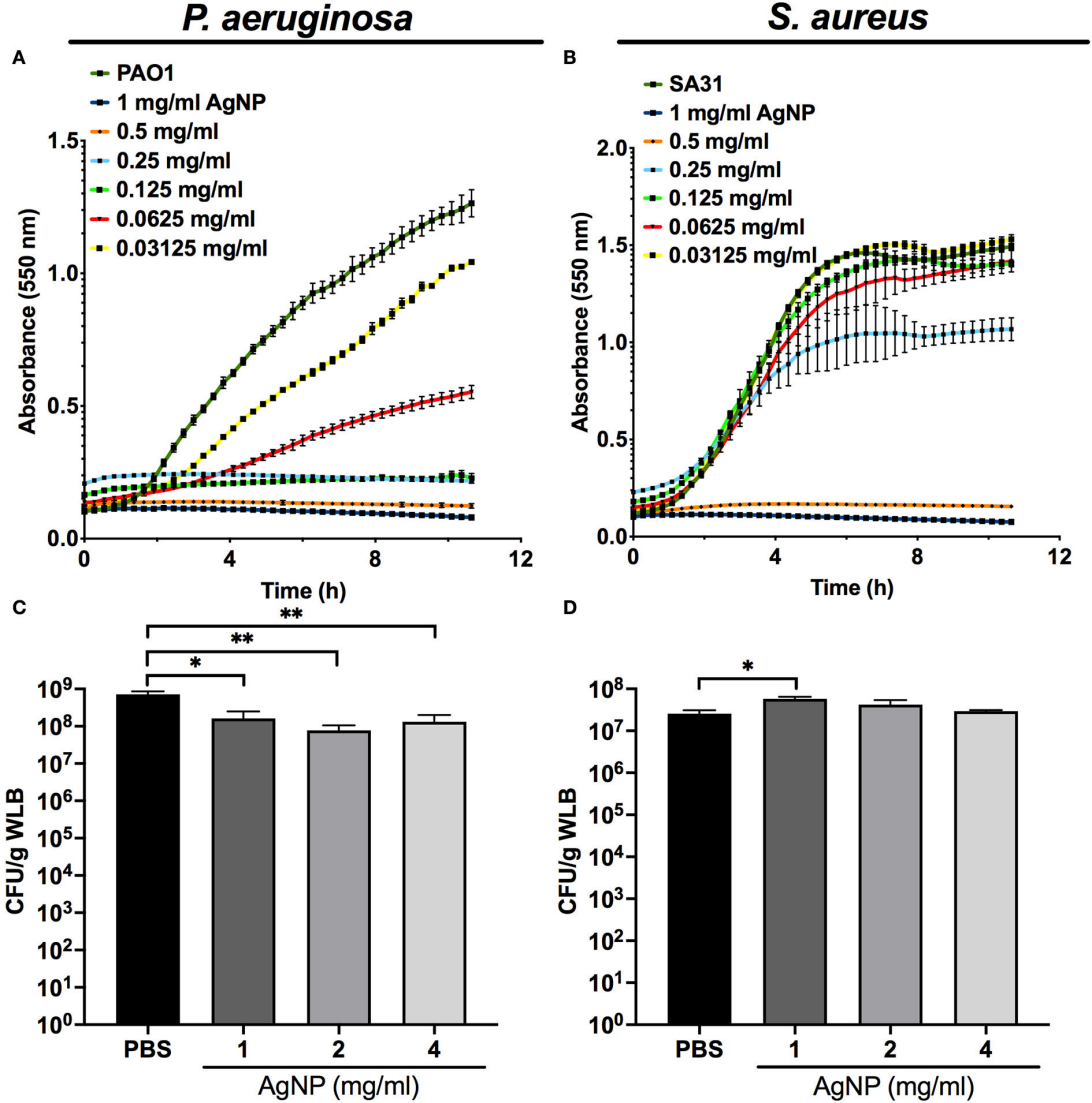
Antimicrobial capacity of silver nanoparticles (AgNP) on *P. aeruginosa* and *S. aureus*. Antimicrobial kinetic assay of silver nanoparticles (AgNP) on *P. aeruginosa* and *S. aureus* planktonic cultures **(A,B)** and dual-species WLB **(C,D)**. Different AgNP concentrations were used to determine the best antimicrobial concentration. The statistical analysis to determine significance was performed using the Student Unpaired *t*-test (*0.01 < *p* < 0.05; **p < 0.01; vs. non-treated WLB).

Our kinetics assays reveal that the silver released from the AgNP had a differential antibacterial effect on planktonic cultures of *P. aeruginosa* and *S. aureus*. The antimicrobial activity of silver was higher against *P. aeruginosa*, whose growth was affected by low concentrations of AgNP (>0.03125 mg/ml) (Figure 3A) compared to the inhibition of *S. aureus*, which needed increased concentrations of AgNP (>0.25 mg/ml) (Figure 3B).

Figures 3C,D shows the antibacterial activity of the silver released from different AgNP concentrations on a WLB antimicrobial assay. When both strains grew together in a WLB, the biofilm structure protected *S. aureus* from the bactericidal action of the AgNP. The AgNP showed a bactericidal effect in WLB against *P. aeruginosa* when using a concentration of at least 1 mg/mL (Figure 3C). However, the tested concentrations did not modify the *S. aureus* cell numbers (Figure 3D).

### Effect of biofilm-dispersing strategies and antimicrobial therapies against *P. aeruginosa* and *S. aureus in vitro* wound-like biofilm model

We tested the ability of four enzymes that target different EPS components to disrupt the matrix structure of a *P. aeruginosa*–*S. aureus* polymicrobial biofilm. The biofilm-dispersing activity of these enzymes was tested using free enzymes or immobilized on AgNP at the same working concentration. In addition, the enzymes were tested alone or with gentamicin and ciprofloxacin to evaluate whether the combined treatment enhanced antibiotic efficacy against the dual-species wound biofilm.

**Figure 4:**
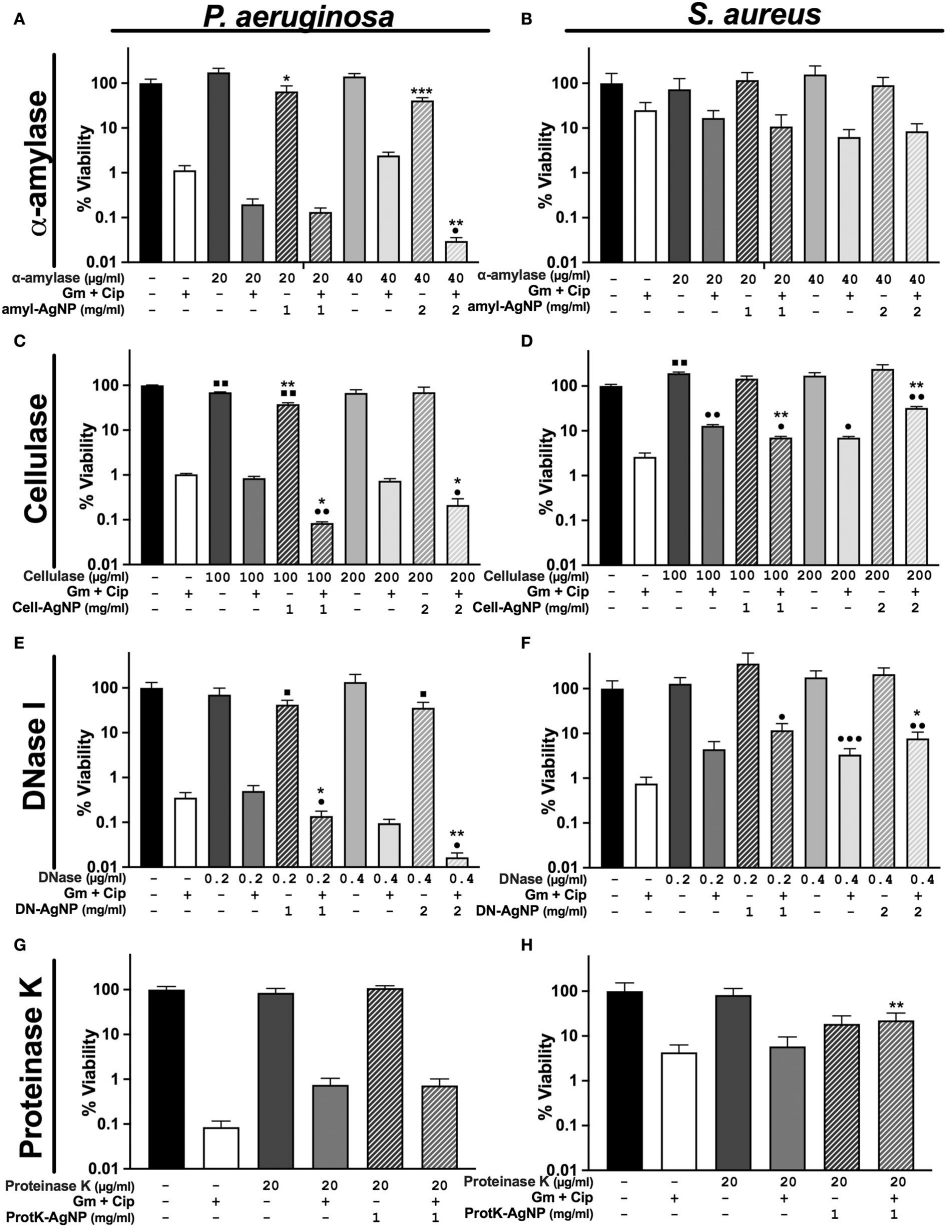
Differential response of antibiofilm treatments on *P. aeruginosa* and *S. aureus* polymicrobial WLB. WLB antimicrobial assays show the effect of the antibiofilm treatments consisting of ciprofloxacin (Cip) and gentamicin (Gm) antibiotics at a final concentration of 13.5 μg/ml each, along with a biofilm-dispersing enzyme in solution or functionalized on AgNP at a final concentration of 1 or 2 mg/ml. The concentration of enzyme indicated above the concentration of AgNP corresponds to the amount of enzyme functionalized on AgNP. Percentage of viability corresponds to counts of CFU on plate from the bacteria in WLB. **(A,B)** α-amylase, **(C,D)**, cellulase **(E,F)**, DNase I, and **(G,H)** proteinase K. In each antimicrobial assay, three biological replicates were used, and two technical replicates were performed for each biological sample. The statistical analysis to determine significance was performed using the Student Unpaired *t*-test (■, 0.01 < *p* < 0.05; ■■, *p* < 0.01, vs. non-treated WLB. ^•^0.01 < p < 0.05; ^••^p < 0.01; ^•••^p < 0.001; vs. antibiotic-treated WLB. *0.01 < *p* < 0.05; **p < 0.01; ***p < 0.001; functionalized AgNP vs. enzyme in solution at the same conditions).

The WLB antimicrobial assays using α-amylase and cellulase showed higher antibiofilm activity against *P. aeruginosa* than against *S. aureus* (Figures 4A–D). Regarding the general efficacy of these two enzymes, their impact on the biofilm was low. However, higher concentrations of α-amylase and cellulase could have been used to test whether the disruptive effect of the enzymes was increased. Besides, the results show that the activity of the immobilized enzyme AgNP was higher than the enzyme in its soluble form. Nonetheless, this outcome could be due to the antimicrobial effect of the silver rather than from the enzyme *per se*, just as is observed in Figure 3.

The use of DNase I combined with antibiotics had a remarkable effect against *P. aeruginosa* (Figure 4E). This effect was significantly higher when the enzyme was immobilized on AgNP and applied with antibiotics. Indeed, we and others already showed the outstanding ability of DNase I to inhibit and disperse *P. aeruginosa* biofilms (Baelo et al., 2015). The use of DNase I against *S. aureus* increased their cell number when using free and immobilized enzymes on AgNP. This increase was probably due to the bacterial disaggregation of *S. aureus* inside the biofilm (Figure 4F). Besides, as mentioned before, DNase I showed the highest stability and activity of all the enzymes immobilized to the AgNP surface. In addition, to study the behavior of single antibiotics and DNase I, we experimented using gentamycin or ciprofloxacin monotherapy with the same concentration of dispersing enzyme (Supplementary Figure S3). We observed that the viability of *P. aeruginosa* was higher with gentamycin than with ciprofloxacin and that its viability decreased when the enzyme DNase I was used. On the contrary, the viability of *S. aureus* was higher when using ciprofloxacin than gentamycin, and the addition of DNase I increased the number of bacterial cells.

Finally, it was observed that none of the different proteinase K concentrations used in the biofilm assay dispersed the biofilm independently of the free or conjugated state of the enzyme (Figures 4G,H). This could be explained by the combination of low AgNP stability and low activity of enzymes immobilized at their surface. Higher enzyme concentrations could have been used to test whether the activity of proteinase K improved.

### Spatial bacterial organization in the *P. aeruginosa*–*S. aureus* mixed *in vitro* wound reflects their different antibiotic resistance

**Figure 5:**
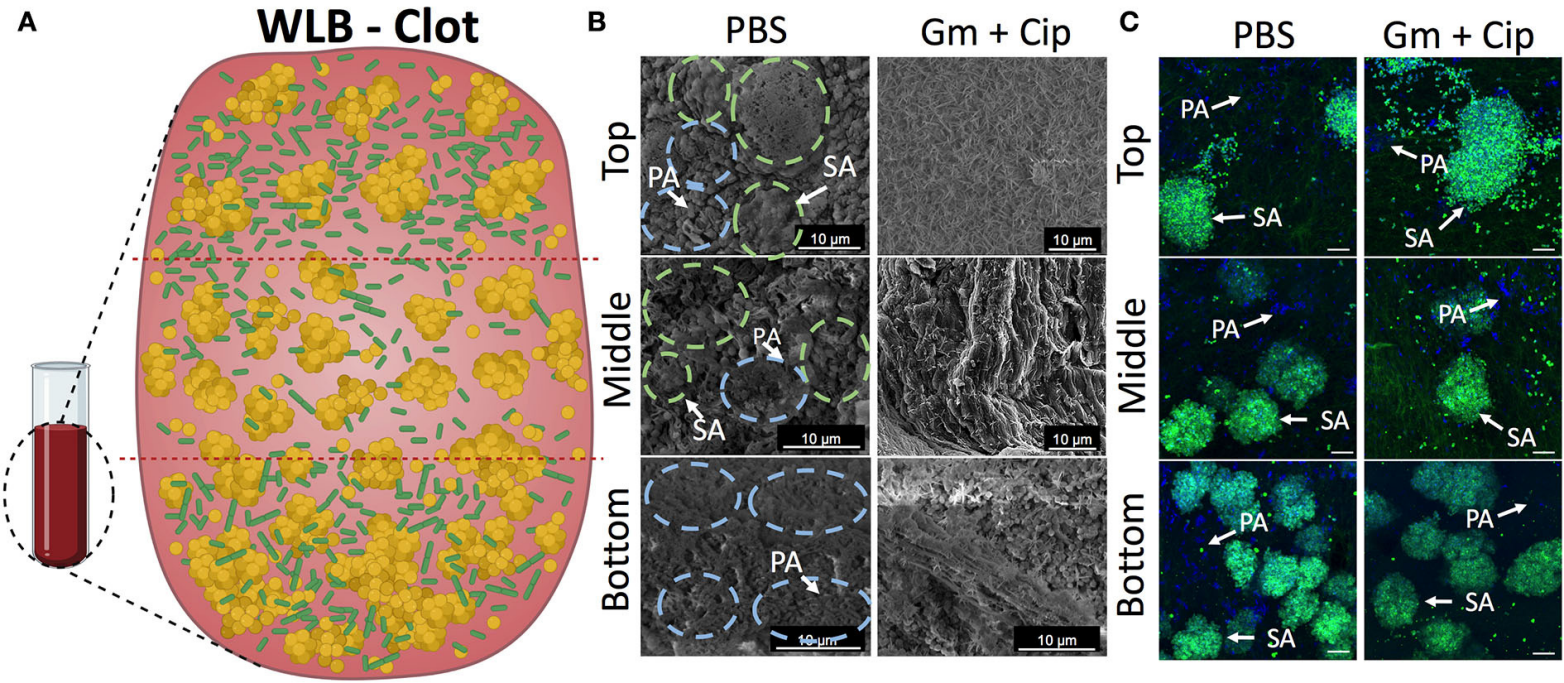
Spatial bacterial distribution in the *in vitro* wound biofilm system before and after antibiotic treatment. Analysis of the transversal parts of the WLB (top, middle, and bottom) **(A)** using FE-SEM **(B)** and CSLM **(C)**. In the FE-SEM images, blue and green dashed circles surround *P. aeruginosa* and *S. aureus* bacterial aggregates, respectively. In CSLM images, *P. aeruginosa* PAO1 (PA) is dyed with DAPI (blue), and *S. aureus* SA31 (SA) is stained with WGA (green). **(A)** Scheme of the three-dimensional WLB colocalization model proposed. **(B)** FE-SEM and **(C)** CSLM representative images of the transversal parts of *P- aeruginosa*–*S. aureus* two-species biofilm in the clots under the effect of a 16-h treatment of ciprofloxacin (Cip) and gentamicin (Gm) and left untreated (PBS). In each region of the clot (top, middle, and bottom), two biological replicates were used, and at least five technical replicates per sample were taken. The scale bar corresponds to 10 μm.

We used the *in vitro* WLB model to analyze the microscopic structure and spatial distribution of *P. aeruginosa* and *S. aureus* polymicrobial wound biofilm. After wound biofilm-forming, we divided the wound biofilm clots into three different transversal parts (top, middle, and bottom) to analyze the localization of the different bacterial species, how they relate to each other, and if each species formed single-species aggregates inside the WLB (see scheme in Figure 5A). As the conditions inside the wound should be different, especially considering oxygen concentration in the different layers of the biofilm, we expected to see differences in the bacterial distribution, such as finding *P. aeruginosa* in the more aerobic parts of the biofilm. Each part was observed with FE-SEM (Figure 5B) and CLSM (Figure 5C). Both microscopy techniques showed that inside the untreated clot (PBS), each bacteria species was forming one-species clusters or microcolonies embedded in a highly dense matrix, which allowed the compartmentation and physical separation of the different bacterial species. The images show that *P. aeruginosa* (PA) [in blue dashed circles in FE-SEM images and dyed with DAPI (blue) in the CSLM images] formed microcolonies located throughout the outside areas, mainly being present in the top and the bottom regions of the clot. However, *S. aureus* (SA) [in green dashed circles in FE-SEM images and dyed with DAPI and WGA (green) in the CSLM images] aggregated compactly, forming clusters placed mainly in the inside part of the clot. The size of the clusters increased as the layers deepened inside the clot, finding the largest *S. aureus* aggregates in the bottom region (Figures 5B,C). The size of the *S. aureus* clusters in each region was quantified and plotted in histograms. The histograms confirmed the cluster distribution observed in the WLB (Supplementary Figure S4).

Since we detected different responses to gentamicin and ciprofloxacin treatments in the wound biofilm environment that directly depended on the bacterial species (Figure 1), we hypothesized that the differential effect would depend on the location and distribution of each bacterium in the wound biofilm environment. The three-dimensional bacterial species organization and matrix composition of the clot might cause differential penetration of the antibiotics in different geographical areas of the biofilm. We used FE-SEM and CLSM to analyze the outcome of the transversal regions of the mixed wound biofilm after the antibiotic treatment. The FE-SEM images (Figure 5B) show that the overall biofilm structural appearance was modified after the antibiotic treatment. The images show that the biofilm matrix was changed completely, becoming a mesh of porous fibrin fibers with low EPS and a matrix with holes that included much bacterial debris. The CSLM (Figure 5C) images show the bacterial viability reductions observed in previous experiments (Figure 1). The combined use of gentamicin and ciprofloxacin resulted in a bacterial reduction load, especially for *P. aeruginosa*, throughout the different traversal parts of the WLB. However, the bacterial reduction load for *S. aureus* seemed less clear.

### DNase I degrade eDNA favoring bacterial dispersion and enhancing antibiotic penetration in the wound-like biofilm

**Figure 6:**
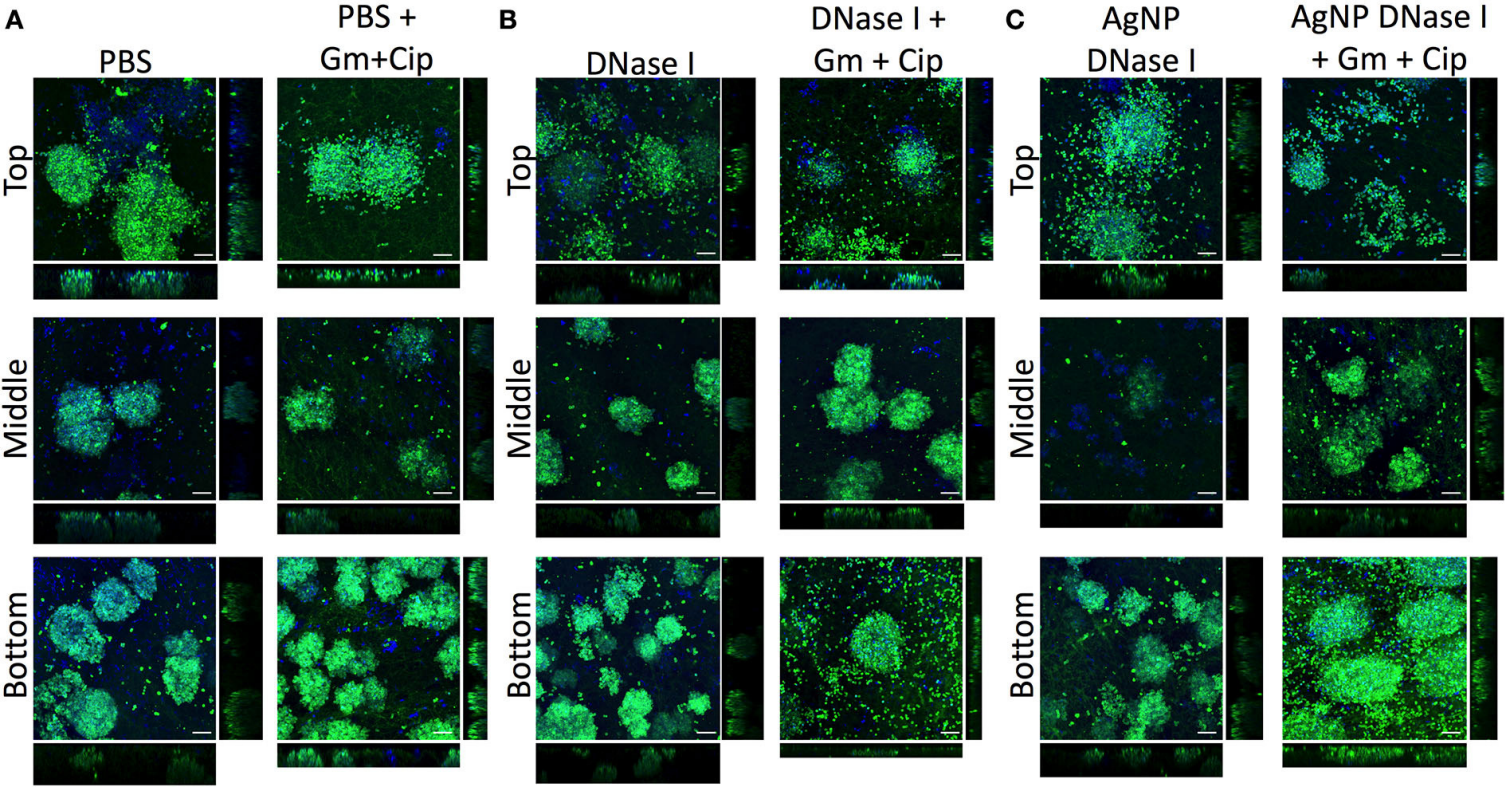
Distribution and colocalization of *P. aeruginosa* PAO1 and *S. aureus* SA31 in the WLB after using DNase I treatment. Analysis of the top, middle, and bottom parts of a wound-like biofilm treated with DNase I alone or combined with gentamicin and ciprofloxacin. **(A)** Gentamicin (Gm) and ciprofloxacin (Cip) treatment, **(B)** gentamicin and ciprofloxacin treatment in combination with soluble DNase I, and **(C)** gentamicin and ciprofloxacin treatment in combination with encapsulated DNase I on silver nanoparticles (AgNP). The Bacterial Viability and Gram Stain Kit differentiated *P. aeruginosa* (blue) and *S. aureus* (green). The scale bars at the bottom-right corner of the images represent 10 μm.

The EPS in the WLB forms a complex structure that helps to protect the bacterial species from different antimicrobials or the host immune response. eDNA is a key element that forms part of the EPS and the structure of the WLB. The antibiofilm assays (Figure 4) showed that the enzyme DNase I increased the antimicrobial activity of combined gentamicin and ciprofloxacin against *P. aeruginosa* when used in combination, especially when the enzyme was immobilized on AgNP. However, the combination of DNase I and antibiotics increased the bacterial cell number of *S. aureus*. Therefore, we used CLSM to analyze the effect of DNase I on the bacterial structure inside the WLB (Figure 6). The first images show the bacterial structure inside the WLB without treatment and after treatment with gentamicin and ciprofloxacin (Figure 6A). It can be seen that each bacterial species (*P. aeruginosa* dyed with DAPI, in blue; and *S. aureus* dyed with DAPI and WGA, in green) was forming one-species microcolonies that were embedded inside the biofilm matrix. After the antibiotic treatment, the cell number of both bacteria was reduced; however, the decrease of *S. aureus* was not as notable. The use of soluble DNase I (Figure 6B) resulted in the breakdown of the microcolonies of both bacteria.

The dissemination was more notable in the clusters of *S. aureus*, as the number of free single cells increased. The cluster dissemination was mainly observed in the top and bottom regions of the WLB, where single *S. aureus* cells were unattached from the primary aggregates. The use of gentamicin and ciprofloxacin mixed with DNase I decreased the viability of both species in the three transversal parts of the WLB (top, middle, and bottom). The decrease of *P. aeruginosa* in the bottom part of the clot was highly notable. In addition, AgNP coated with DNase I boosted the bacterial disaggregation from their microcolonies (Figure 6C). A similar outcome was observed in the *S. aureus* clusters. The dispersion observed after the use of DNase I-coated AgNP was higher compared to the soluble DNase I. The use of AgNP almost completely broke down the compact *S. aureus* clusters in the top region, and the number of *S. aureus* single cells found in the bottom region increased. The microcolony dissemination was enhanced when the enzyme was combined with antibiotics. The combination of enzyme-antibiotics reduced the total bacterial cell number, being more evident in *P. aeruginosa*.

**Figure 7:**
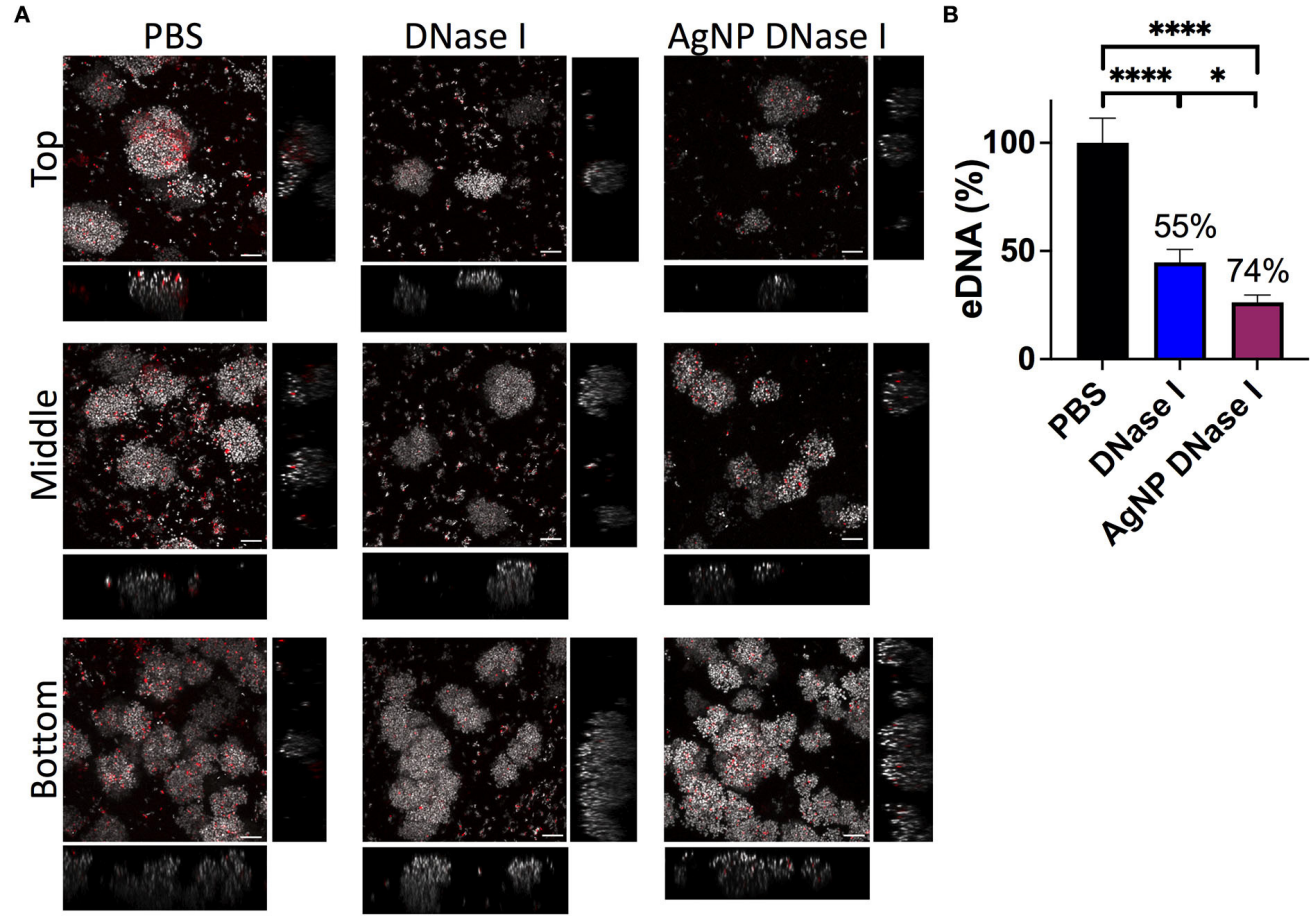
eDNA in the WLB. eDNA analysis in an untreated clot (PBS) and treated with DNase I in a soluble form or encapsulated in a silver nanoparticle (AgNP). **(A)** Presence of eDNA in the top, middle, and bottom parts of the WLB. The dye SYTO60 (gray) stains the inner DNA of the bacteria; the dye TOTO-1 (red) stains the eDNA of the samples. Images were taken at a 63x magnification, and the scale bars represent 10 μm. **(B)** Percentage of eDNA present in the untreated clot and the clot treated with DNase I. Numbers above each bar indicate the percentages of reduction of eDNA comparing treated to untreated samples. The statistical analysis was performed using the Student Unpaired *t*-test (*p < 0.05; ****p < 0.0001).

In addition, we deeply analyzed the eDNA present in the WLB with CLSM using an untreated clot and a clot treated with DNase I and AgNP coated with DNase I (Figure 7). The images show that the eDNA present in the WLB (stained with TOTO-1 in red) notably decreased when the clot was treated with DNase I (Figure 7A). It seems that eDNA degradation was more efficient where fewer bacteria were present, such as in the top and middle parts of the clot. We quantified the eDNA present in each sample (PBS, DNase I, and AgNP-DNase I) and calculated the percentage of eDNA reduction in the WLB after the treatments (Figure 7B). We determined that eDNA significantly decreased in the clot after treatment with DNase I in its soluble form and immobilized in AgNP by 55 and 74%, respectively. Moreover, the reduction observed when using AgNP-DNase I was significantly higher than the soluble and non-encapsulated DNase I.

## Discussion

A particular biofilm's spatial structure and composition determine its pathogenicity and therapeutic responses. Thus, we deepened into an *in vitro* wound-like biofilm that contained a stable community of two of the most frequent bacteria found in wounds, *P. aeruginosa* and *S. aureus*. We explored how gentamicin and ciprofloxacin, two common broad-spectrum antibiotics, affected the viability of *P. aeruginosa* and *S. aureus* when growing as a dual-species wound biofilm. We expected a reduced antibiotic susceptibility compared with monoculture biofilms since previous investigations already reported this behavior in the wound biofilm model (DeLeon et al., 2014). Besides, the antibiotics' effect on the species dynamics has not been previously analyzed. Here, we demonstrate that gentamicin and ciprofloxacin shift the population distribution in the wound biofilm environment, driving a particular bacterial species to outcompete the other. Whereas, *P. aeruginosa* remained unaffected by a 24-h treatment with gentamicin, its viability was reduced by 3.5 logs when ciprofloxacin was used.

On the contrary, *S. aureus* viability decreased by 1.5 logs when using gentamicin and nearly unchanged when using ciprofloxacin (Figure 1). The increased gentamicin tolerance of *P. aeruginosa* in the wound biofilm may be due to alginate, one of the main components of the EPS matrix, among other factors (DeLeon et al., 2014). The mechanism by which ciprofloxacin lacked activity against *S. aureus* in the wound biofilm has not yet been investigated. Still, other investigations have detected that *S. aureus* showed low susceptibility against ciprofloxacin in a multispecies wound biofilm in which *P. aeruginosa* is co-cultured (DeLeon et al., 2014; Townsend et al., 2017). Recently, it was described that *S. aureus* was more tolerant to ciprofloxacin due to the inhibition of its respiration, which reduced ATP levels (Radlinski et al., 2017). However, ciprofloxacin possesses low solubility and permeability, leading to restricted diffusion into *S. aureus* cells. Whether *S. aureus* enhanced tolerance to ciprofloxacin due to *P. aeruginosa*, a restricted penetration into the wound biofilm structure, or both remains unexplored.

These outcomes show that only the use of both antibiotics combined had an additive effect and reduced the viability of *P. aeruginosa* and *S. aureus* by 3.2 logs. Other studies have shown that therapies combining different antibiotics are useful tools for treating biofilm-related infections (Ciofu et al., 2017). The response to antibiotic chemotherapies may be considered when treating biofilm infections since choosing the inappropriate antibiotic could lead to an unbalanced microbial population and enhance the growth of the more pathogenic bacteria. The dual-antibiotic therapy reduced the viability of both bacteria, improved the overall antibiotic efficacy, and maintained a more balanced population distribution, improving infection resolution.

One of the main biofilm treatment problems is some antibiotics' poor penetration. Therefore, there is an urgent need to find high penetrability antimicrobial compounds against chronic wound infections. Silver-containing creams are extensively used to treat burns and wounds as they reduce the viability of *P. aeruginosa* and *S. aureus* by 99% (Wu et al., 2014; Zhou et al., 2016). AgNP is an outstanding therapy against wound infections due to its enhanced inhibitory effect against bacteria and its ability to encapsulate large amounts of enzyme (Radulescu et al., 2016; Kumar et al., 2018). In this study, we produced AgNP with enzymes immobilized on their surface. We observed that the enzyme immobilization reduced the particle size and changed its zeta potential, increasing its penetrability (Figure 2). We determined the antimicrobial activity of AgNP on *P. aeruginosa* and *S. aureus* planktonic cultures and in WLB. The use of AgNP at low concentrations reduced the viability of *P. aeruginosa* and *S. aureus* when growing on planktonic cultures. When AgNP was used against a dual-species biofilm, the viability of *P. aeruginosa* decreased 10 times, whereas the total cell number of *S. aureus* was maintained (Figure 3). This result indicates that the AgNP cannot eliminate the bacteria embedded in the biofilm, probably because it does not penetrate completely into the EPS matrix.

Here, we explored the use of α-amylase, cellulase, DNase I, and proteinase K to disrupt EPS components that may be relevant in the polymicrobial wound model. In addition, we hypothesized that the viability of *P. aeruginosa* and *S. aureus* would decrease more using the enzymes immobilized on AgNP than in their free soluble form due to the silver antibacterial effect already observed (Figure 3).

α-amylase and cellulase have previously been shown to disperse the EPS in wound biofilms; thus, we hypothesized that these enzymes were promising to be used in combination therapies (Fleming et al., 2017; Fleming and Rumbaugh, 2018; Redman et al., 2020). Our results indicate that the viability of *P. aeruginosa* decreased when using α-amylase and cellulase, and this decrease was higher when the enzymes were immobilized on AgNP (Figures 4A,C). However, the viability of *S. aureus* increased when using these enzymes (Figures 4B,D), possibly due to the enzymatic dispersion of cells from the defined cluster increasing the bacterial cell number counts. Therefore, we believe that α-amylase and cellulase have an excellent potential to be used as antibiofilm compounds in combination therapies. Fleming et al. (2020) already observed the ability of glycoside hydrolase treatments to break the bacterial clusters of *P. aeruginosa* and *S. aureus*.

DNase I has been widely tested as an antibiofilm agent because of its ability to degrade eDNA, which is the major component of most single-species biofilms formed by *P. aeruginosa* and *S. aureus* (Sugimoto et al., 2018). Our results indicate that DNase I was the most promising enzyme to be used because *P. aeruginosa* viability was significantly reduced when used in combination with gentamicin and ciprofloxacin. This decrease was more remarkable when the enzyme was immobilized on AgNP. This observation lines with Modak and Fox (1973). They observed that silver binds to DNA, interfering with bacterial replication, so the use of DNase I attached to AgNP may enhance the degradation of eDNA (Figure 4E) and possibly inhibit bacterial proliferation.

On the contrary, the number of viable cells of *S. aureus* increased when using DNase I, especially when the enzyme was immobilized. eDNA has been described to accumulate mainly in *S. aureus* aggregates in a wound biofilm model like we employed (Kucera et al., 2014). We hypothesized that the aggregated bacterial cells were disseminated when the eDNA was degraded, increasing the number of bacterial cells observed in the biofilm viability test (Figure 4F). This indicated that eDNA might have a role in reducing antibiotic susceptibility on the *S. aureus* aggregates surrounded by eDNA. These results support the idea that DNase I may be a strong antibiofilm compound, especially when linked to AgNP. Thus, the combination therapy of DNase I with antibiotics may be an important strategy for the future. In addition, the results using single antibiotics as monotherapy showed that using both antibiotics simultaneously is a better approach to target both bacterial species inside the wound.

Proteinase K is an unspecific serine endopeptidase that possesses hydrolytic activity in a wide range of protein substrates. Since proteinaceous fibrin fibers mainly form the matrix derived from the wound environment, protein degradation may improve the destabilization of the biofilm. However, we could not demonstrate that proteinase K enzymatic treatment improves the antibiotic efficacy on the wound biofilm by increasing antibiotic activity against the two bacterial species (Figures 4G,H). This outcome may be due to the low AgNP stability and low activity of the enzyme (Table 1).

It is essential to highlight that the high heterogeneity observed for *S. aureus* was possible because the bacterial clusters observed inside the wound also present high-sizeheterogenicity. Depending on the efficacy of dispersing bacterial aggregations, the releasing bacteria would form viable counts (increasing CFU). Thus, the data obtained were more heterogeneous than those from *P. aeruginosa*.

Although these treatments seemed promising, it would probably be necessary to combine several dispersing enzymes to eliminate the biofilm, as the EPS degrading efficiency depends on the EPS composition (Redman et al., 2021).

In this work, we also studied the interaction, colocalization, and synergic relationship between *P. aeruginosa* and *S. aureus* in a dual-species wound biofilm. In concordance with other authors that have analyzed *in vivo* and *in vitro* polymicrobial wound biofilms (Kirketerp-Moller et al., 2008; Fazli et al., 2009; Dalton et al., 2011; Woods et al., 2012; Kucera et al., 2014; Brackman et al., 2016), we found that bacteria segregated in single-species aggregates are covered by matrix depositions. Despite a detailed description of the structure and bacterial distribution that has not been depicted before, some of these investigations analyzed the distribution of *S. aureus* and *P. aeruginosa* based on their relative distance to the wound surface. However, whereas some authors indicate that *S. aureus* aggregates locate closer to the surface and *P. aeruginosa* penetrates deeply in the wound bed (Kirketerp-Moller et al., 2008; Fazli et al., 2009), others differ and found *P. aeruginosa* near the surface and more aerobic areas (Dalton et al., 2011; Woods et al., 2012; Kucera et al., 2014). In this study, we analyzed different transversal areas of the wound biofilms and represented for the first time an overall distribution scheme. We observed that *P. aeruginosa* bacterial aggregates extend throughout the biofilm but with a preference for the outer regions (top and bottom areas) (Figure 5A), which may be due to a *P. aeruginosa* preference for the aerobic or completely anaerobic environments like other investigations indicated (Woods et al., 2012; Brackman et al., 2016). As for *S. aureus*, bacterial aggregates were located in the central parts of the biofilm-forming compact bacterial aggregates, being the bottom part where the most extensive aggregates were found, and surrounded by a dense matrix layer, probably following the abscess formation process in which the created fibrin meshwork protects bacteria from the immune cell attack and prevents antibiotic penetration (Vanassche et al., 2013; Dastgheyb et al., 2015). We think these results may be relevant when designing specific antibacterial treatments against wound biofilms since the bacterial location and physical features of a particular biofilm in the infection site may influence the effect of local antimicrobials and their ability to reach bacterial cells.

Besides affecting bacterial viability and overall species distribution within the biofilm (Figures 5B,C), decreasing the amount of *P. aeruginosa* cells, and increasing the number of free *S. aureus* cells, antibiotic treatment notably modified the overall wound biofilm structure and matrix composition (Figure 5B). Gentamicin and ciprofloxacin increased the porosity of the biofilm through changes in the EPS matrix production, as other antimicrobials do (Schilcher et al., 2016; Andre et al., 2019). We believe that bacteria may alter the composition of the matrix as an adaptive mechanism to enhance protection against antibiotics.

The overall species distribution within the biofilm changed after using DNase I. Other authors have already observed the outstanding ability that DNase I presents, breaking the eDNA of the EPS in biofilms (Baelo et al., 2015). Our results indicate that the degradation of the eDNA triggered the dissemination of single cells from the bacterial aggregates (Figure 6). It seemed that the number of free cells from *S. aureus* was higher than those from *P. aeruginosa*. These results would indicate that the clusters of *S. aureus* stuck together using eDNA, and when it was degraded, the cells were disaggregated from the cluster. The cell dissemination was more notable on the top and bottom parts of the WLB, especially when the DNase I was immobilized on AgNP (Figure 6). We hypothesize that bacterial aggregates protect single bacteria against antibiotics, being disseminated cells more susceptible to them. The use of enzymes, especially DNase I, that favor cell dispersion from the bacterial clusters would increase the number of disseminated bacteria and thus their susceptibility (Figure 4).

Finally, in our results, we observed and quantified a significant reduction of eDNA inside the WLB after using DNase I (Figure 7). eDNA reduction seemed more efficient on the top and bottom parts of the WLB, just as is observed in Figure 6. Besides, the graph showed that eDNA reduction was significantly higher when using DNase I immobilized on AgNP (Figure 7B). This increased effect could be explained due to the DNA binding proprieties of the silver ions present on the nanoparticles (Modak and Fox, 1973). Silver ions would facilitate the binding of DNase I to the eDNA and enhance its degradation, the dissemination of the cells, and finally, the bacterial elimination with antibiotics.

The question of applying the functionalized AgNP with antibiotics for *in vivo* wound treatment should be addressed for future perspectives. Other authors have already dealt with this issue and pointed out new insights in the field (Blanco-Fernandez et al., 2021; Ndlovu et al., 2022).

## Conclusion

Our findings may provide valuable information about how biofilm's architecture and matrix composition, shaped by both microorganisms and the environment, may influence antibiotic susceptibility and population dynamics in a multispecies wound biofilm. By determining the effect of matrix-degrading enzymes, we have demonstrated that polysaccharides and eDNA play a key role in wound biofilm stability. Furthermore, the colocalization and species distribution studies have provided a broader vision of the species interrelation inside the wound biofilm. Altogether, these results may help to improve current antibacterial therapies in wound infections. Future studies combining several enzymes should be carried out to test whether it increases the enzymes' dispersing activity and the antibiotics' antimicrobial effect. In addition, these treatments should be tested against multidrug-resistant bacteria and clinically isolated bacteria to assure their efficacy and possible future clinical use.

## Data availability statement

The raw data supporting the conclusions of this article will be made available by the authors, without undue reservation.

## Author contributions

AR-C, AB, SH, and NB-C performed biological assays and wrote the manuscript. MV performed the NP synthesis and characterization. ET directed the research, revised the experimental data, and wrote the manuscript. The manuscript was written through the contributions of all authors. All authors have approved the final version of the manuscript.

## Funding

This study was partially supported by grants from the MCIN/AEI/10.13039/501100011033 and ERDF A way of making Europe (RTI2018-098573-B-100 and PID2021-125801OB-100), the CERCA program and *AGAUR-Generalitat de Catalunya* (2017SGR-1079), the European Regional Development Fund (FEDER), and Catalan Cystic Fibrosis association and Obra Social La Caixa. AR-C is thankful to MCIN/AEI/10.13039/501100011033 and ESF Investing in your future, for her financial support through FPI (PRE2018-083709).

## Conflict of interest

The authors declare that the research was conducted in the absence of any commercial or financial relationships that could be construed as a potential conflict of interest.

## Publisher's note

All claims expressed in this article are solely those of the authors and do not necessarily represent those of their affiliated organizations, or those of the publisher, the editors and the reviewers. Any product that may be evaluated in this article, or claim that may be made by its manufacturer, is not guaranteed or endorsed by the publisher.
