# Supplementary material for: 3D spatial organization and improved antibiotic treatment of a Pseudomonas aeruginosa–Staphylococcus aureus wound biofilm by nanoparticle enzyme delivery
Source: Front Microbiol. 2022 Nov 16;13:959156. doi: 10.3389/fmicb.2022.959156 (PMC9708873; doi:10.3389/fmicb.2022.959156)
Supplement: Supplementary file 1 [file Data_Sheet_1.PDF]

3D spatial organization and improved antibiotic treatment of a *Pseudomonas aeruginosa – Staphylococcus aureus* wound biofilm by nanoparticle enzyme delivery

Alba Rubio-Canalejas[1], Aida Baelo[1], Sara Herbera[1], Núria Blanco-Cabra[1,3], Marija Vukomanovic[2] and Eduard Torrents[1,3*]

[1]Bacterial infections and antimicrobial therapies group, Institute for Bioengineering of Catalonia (IBEC), The Barcelona Institute of Science and Technology (BIST), Barcelona, Spain.
[2]Advanced Materials Department, Institute Jozef Stefan, Ljubljana, Slovenia
[3]Microbiology Section, Department of Genetics, Microbiology and Statistics, Faculty of Biology, University of Barcelona, Barcelona, Spain.

Running title: Wound biofilm treated with nanoparticles. (5 words max)

***Corresponding author:**
Dr. Eduard Torrents, Bacterial infections and antimicrobial therapies group, Institute for Bioengineering of Catalonia (IBEC), Baldiri Reixac 15-21, 08028, Barcelona, Spain; e-mail: etorrents@ibecbarcelona.eu

FIGURE S1. Optimization of gentamicin and ciprofloxacin concentration in dual-species WLB. *P. aeruginosa* and *S. aureus* bacterial cell number in the clot (CFU/g) after 24 h treatment of different concentrations (µg/ml) of gentamicin and ciprofloxacin used simultaneously. The statistical analysis to determine significance between the untreated control (0 µg/ml) and the different antibiotic concentrations was performed using the Student Unpaired *t*-test (*, $p < 0.05$).

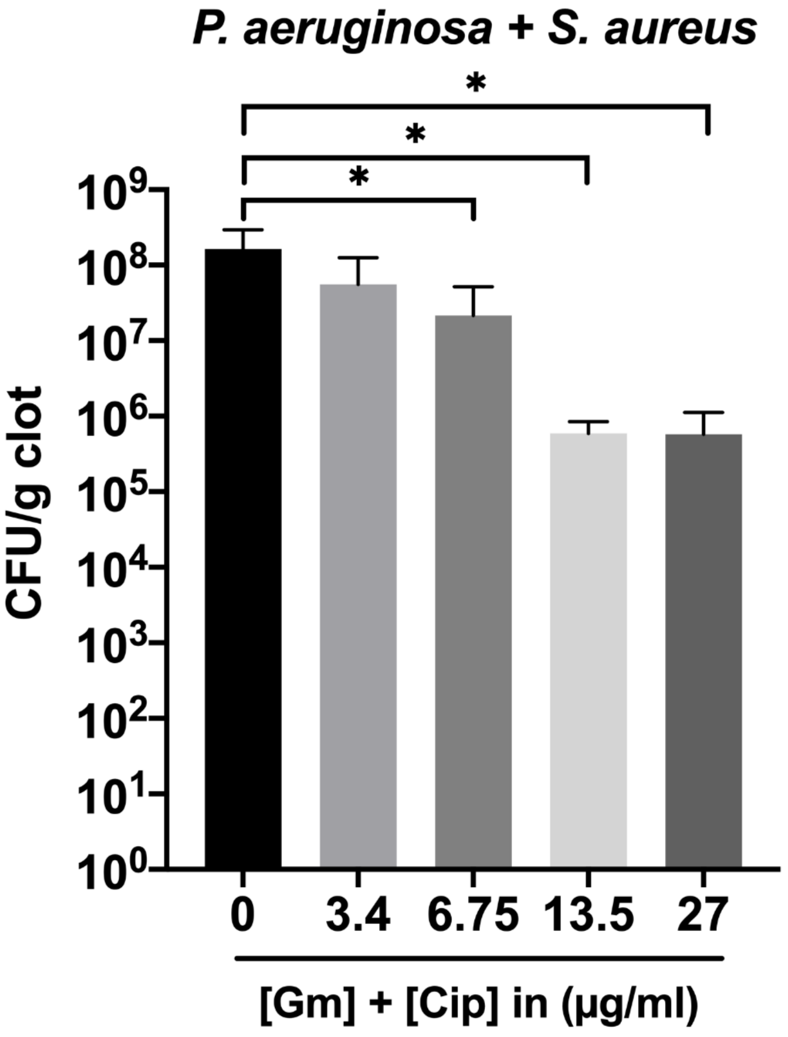

FIGURE S2. Growth of *P. aeruginosa* and *S. aureus* on different media. Growth of *P. aeruginosa* PAO1 and *S. aureus* SA31on LB agar containing 2 mg/ml of crystal violet and TSA containing 7.5 % (w/v) of NaCl.

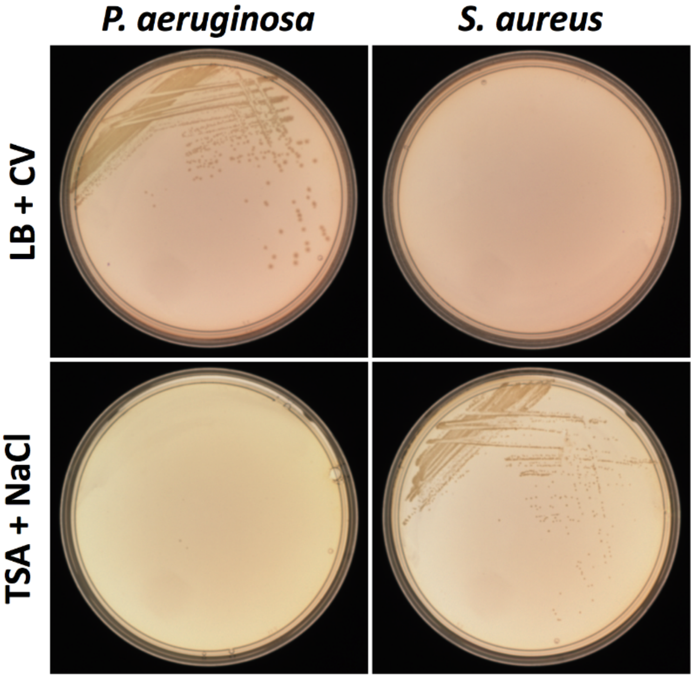

FIGURE S3. Antimicrobial assays on *P. aeruginosa* and *S. aureus* polymicrobial WLB. Effect of the antibiofilm treatments consisting of single use of gentamicin (Gm) or ciprofloxacin (Cip) at a final concentration of 13.5 µg/ml each, along with DNase I. Percentage of viability corresponds to counts of CFU on plate from the bacteria in WLB. The statistical analysis to determine significance was performed using the Student Unpaired *t*-test (*, $p < 0.05$; **, $p < 0.01$).

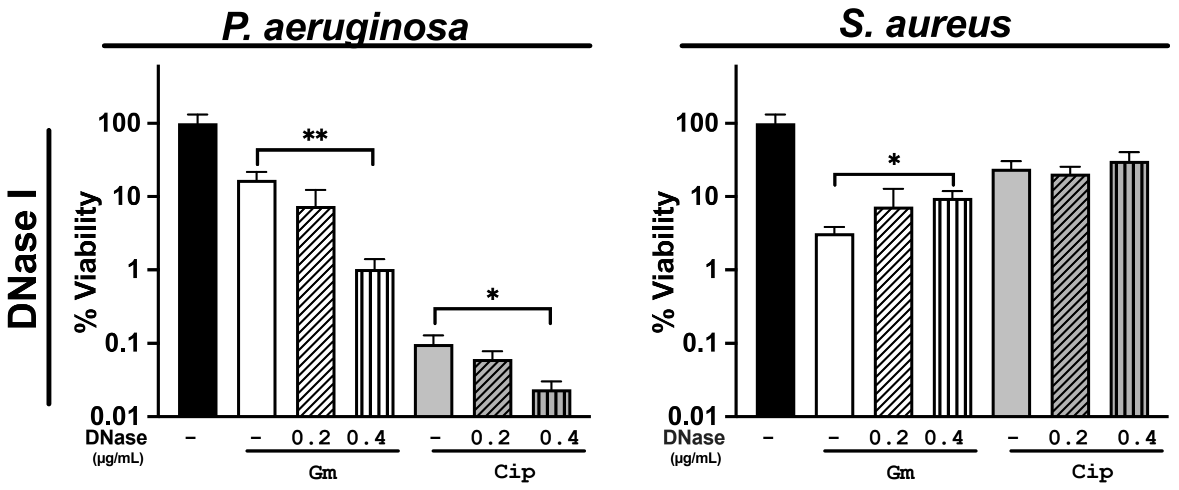

FIGURE. S4. **Histograms of the area of the *S. aureus* clusters to evaluate their distribution in the different traversal parts of the clot (top, middle, and bottom).**

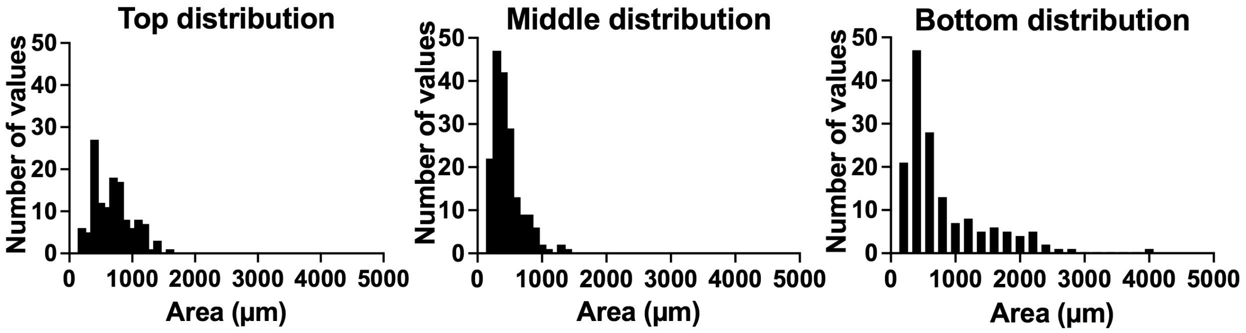

FIGURE S5. Enlarged CSLM images from the wound biofilm. The figures correspond to the Figures 5 and 6 from the main text.

Figure 5:

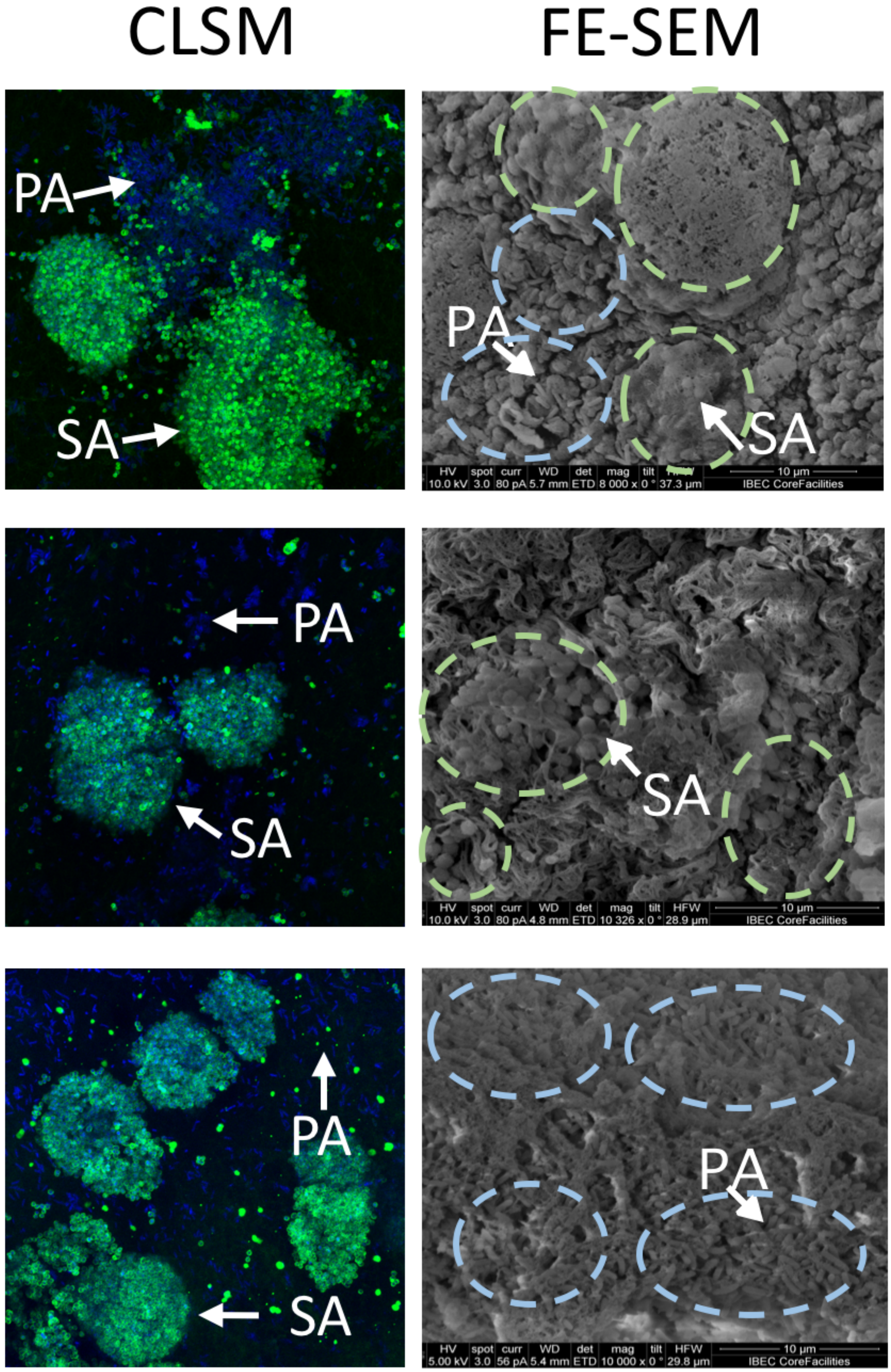

Figure 6:

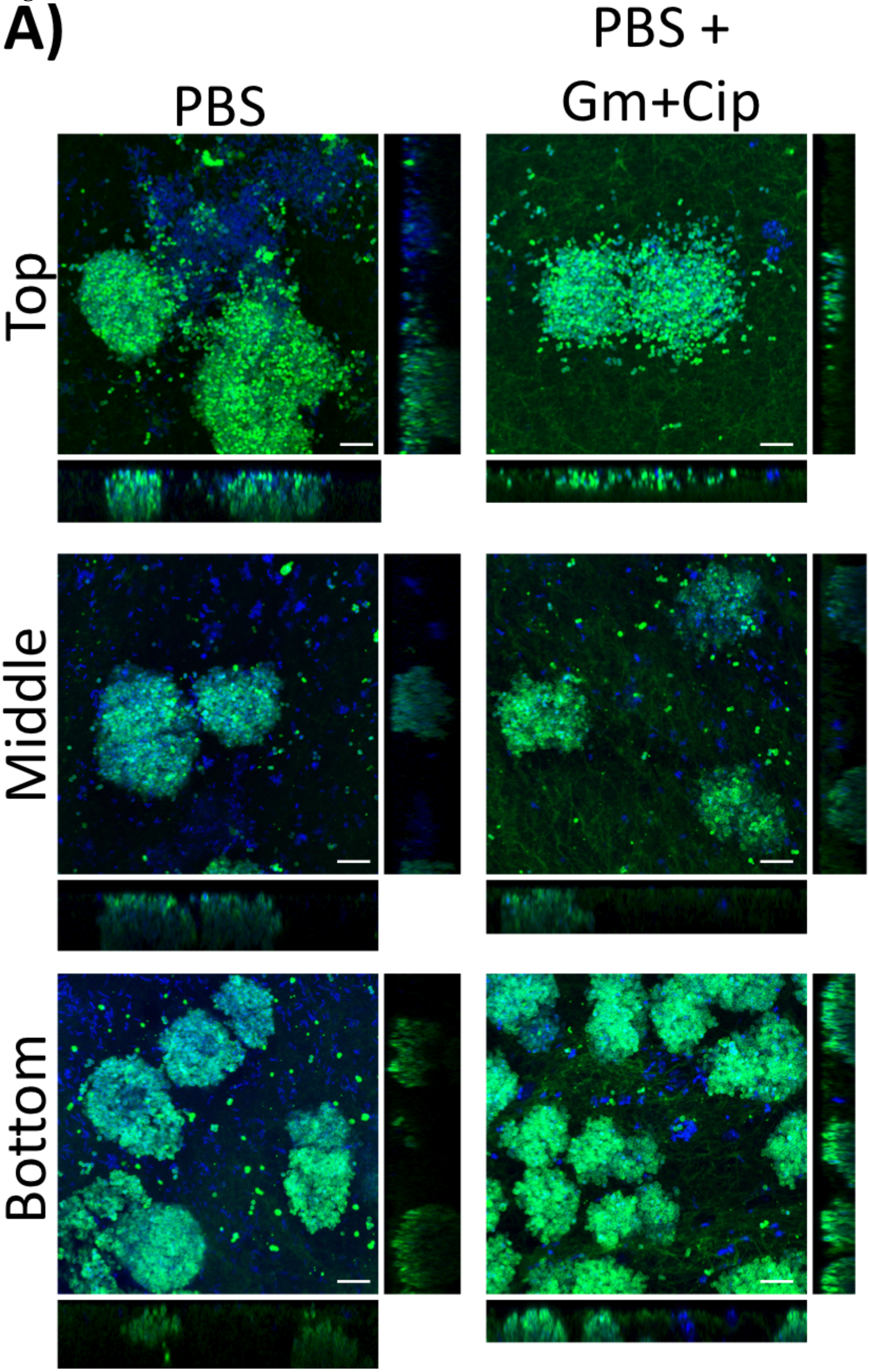

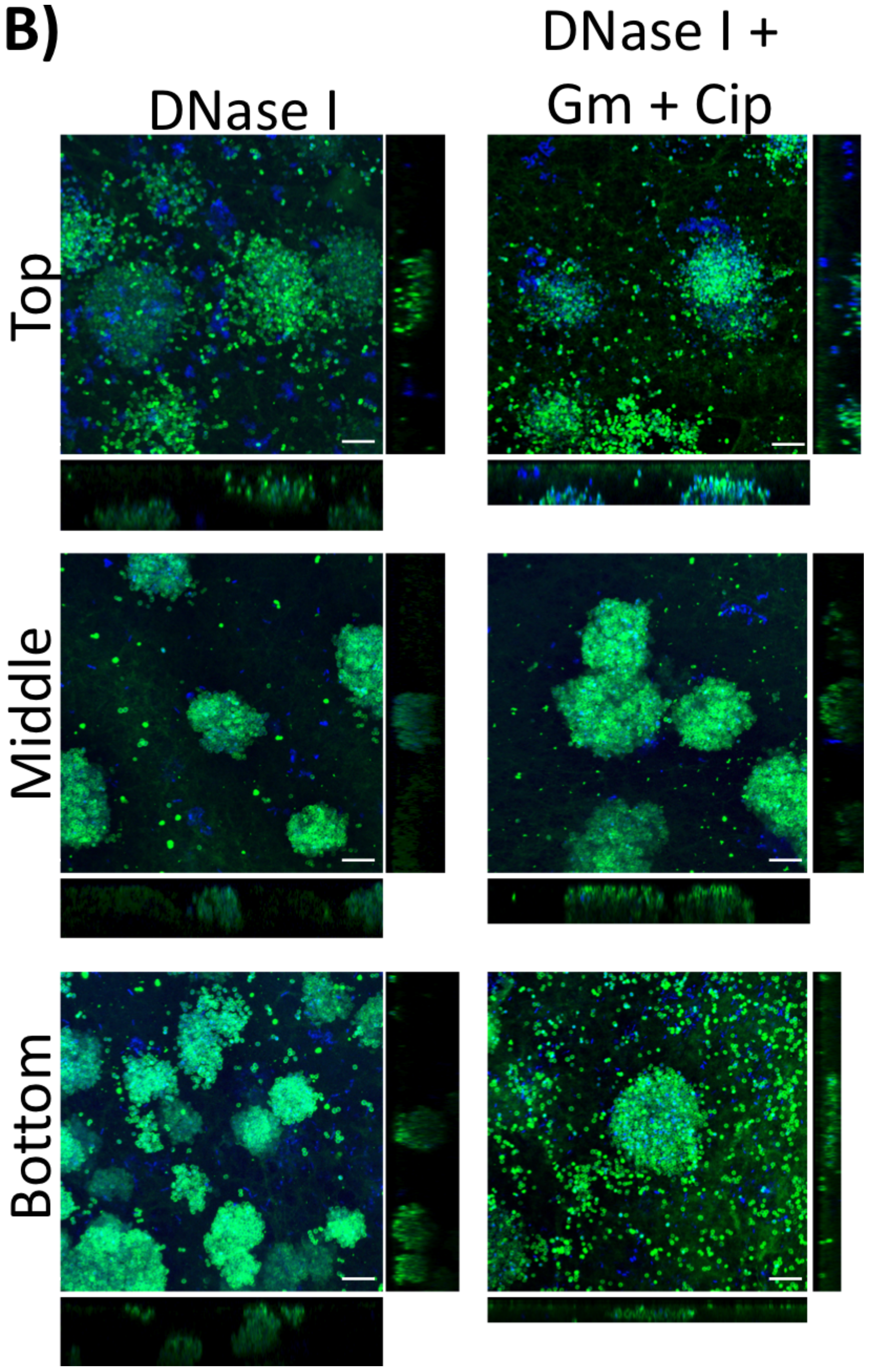
B)
DNase I
DNase I +
Gm + Cip
Top
Middle
Bottom

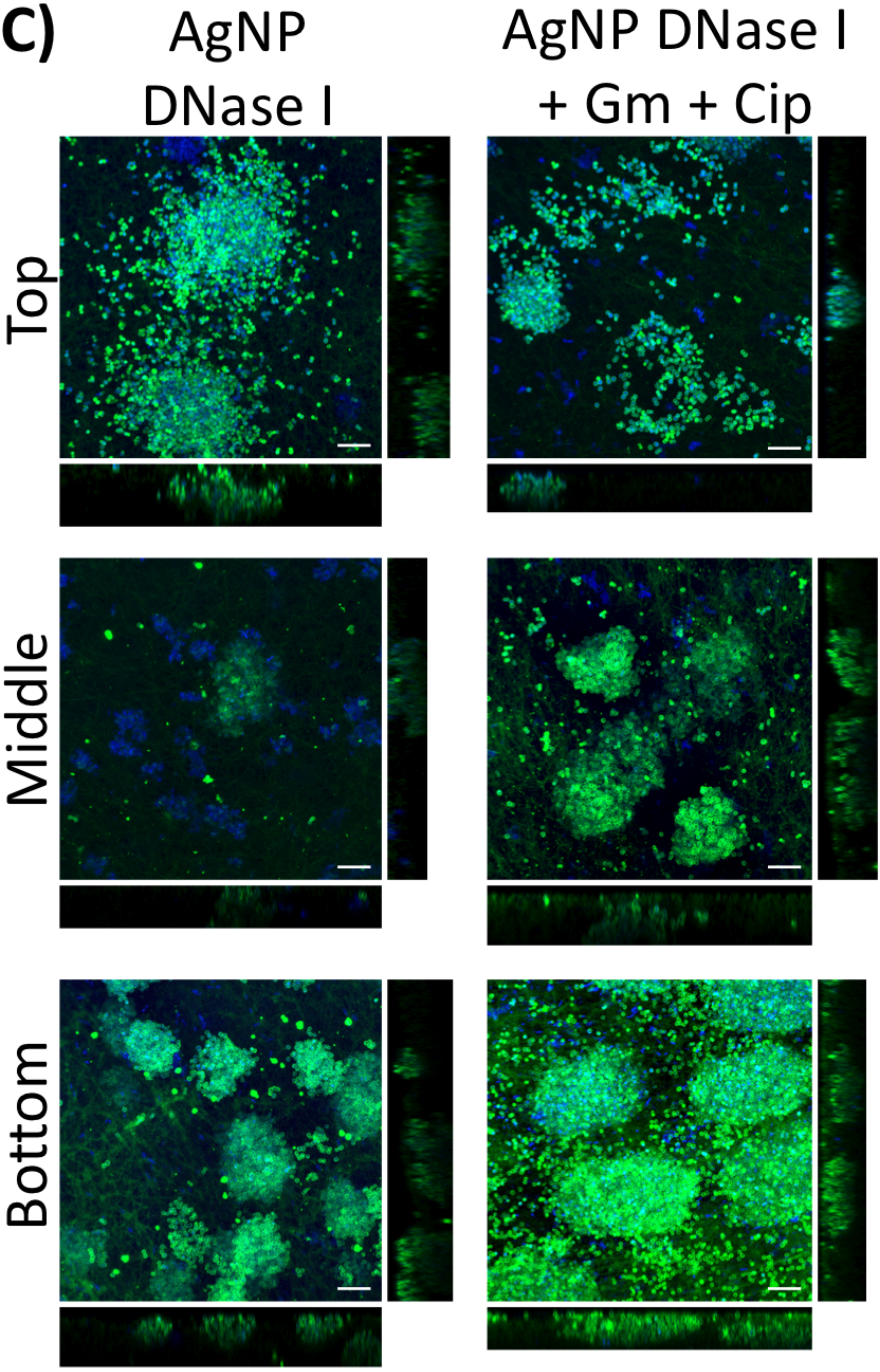
C)
AgNP DNase I
AgNP DNase I + Gm + Cip
Top
Middle
Bottom
